# Liquid chromatography/mass spectrometry based detection and semi‐quantitative analysis of INSL5 in human and murine tissues

**DOI:** 10.1002/rcm.7978

**Published:** 2017-11-06

**Authors:** R.G. Kay, S. Galvin, P. Larraufie, F. Reimann, F.M. Gribble

**Affiliations:** ^1^ Metabolic Research Laboratories Institute of Metabolic Science, Addenbrooke's Hospital Hills Road Cambridge CB2 0QQ UK

## Abstract

**Rationale:**

Insulin‐like peptide 5 (INSL5) is a hormone produced by enteroendocrine L‐cells in the colon that has recently been implicated in the control of metabolic homeostasis. However, research into its physiology has been hindered by the reported unreliability of commercially available immunoassays and additional detection assays would benefit this emerging field.

**Methods:**

Peptides from purified murine L‐cells and homogenates from both human and mouse colonic tissues were extracted by precipitating larger proteins with acetonitrile. Untargeted liquid chromatography/tandem mass spectrometry (LC/MS/MS) analyses, followed by database searching, were used to detect and identify various INSL5 gene derived peptides and characterise their precise sequence. A similar approach was developed to quantify INSL5 levels in primary intestinal culture supernatants after purification and concentration by solid‐phase extraction.

**Results:**

Mass spectral analysis of purified enteroendocrine cells and tissue homogenates identified the exact sequence of A and B chains of INSL5 endogenously expressed in L‐cells. Differences in the endogenously processed peptide and the Swissprot database entry were observed for murine INSL5, whereas the human sequence matched previous predictions from heterologous expression experiments. INSL5 was detected in the supernatant of human and mouse primary colonic cultures and concentrations increased after treatment with a known L‐cell stimulus.

**Conclusions:**

The first LC/MS/MS‐based method capable of the detection and semi‐quantitative analysis of endogenous INSL5 using MS‐based techniques has been demonstrated. The methodology will enable the identification of stimulants for INSL5 secretion from murine and human primary colonic epithelial cultures.

## 1 INTRODUCTION

**Figure 1:**
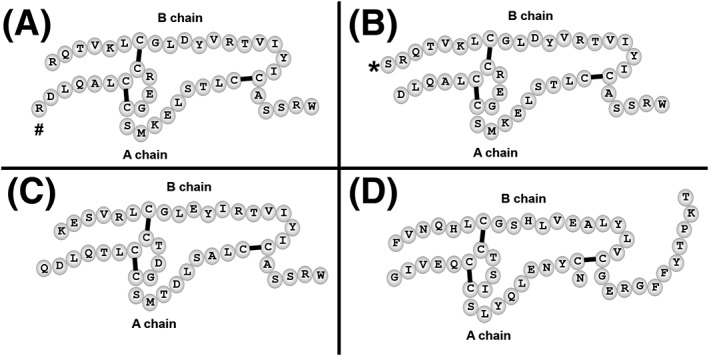
Sequences and disulphide‐bond positions of murine and human mature INSL5 peptides compared to human insulin. A, Murine INSL5 protein as designated by Swissprot, # denotes the additional arginine residue compared to the Phoenix peptide. B, Structure of the Phoenix murine INSL5 peptide, * denotes the additional serine residue compared to the Swissprot sequence in A. C, Structure of human INSL5 as designated by Swissprot. D, Structure of human insulin for comparison

Insulin‐like peptide 5 (INSL5) is a member of the insulin/relaxin gene family sharing a common structural signature comprised of an A and B chain with a conserved cysteine bridging pattern (Figure 1).^1^ INSL5 is expressed at highest levels in the colon of both mice and humans, with lower expression found for example in thymus and testis^1^, ^2^ and within the colon it is expressed in enteroendocrine L‐cells,^3^ which also secrete glucagon‐like peptide‐1 (GLP‐1)^4^ and peptideYY (PYY).^4^ Liu and collaborators showed that INSL5 stimulates the G‐protein coupled receptor RXFP4 (GPR100 or GPCR142) but no precise physiological function of the peptide was initially identified.^5^ Since then INSL5 has been implicated in the regulation of metabolism, either by affecting glucose tolerance through an impact on insulin production,^6^ insulin secretion^7^ or the regulation of hepatic glucose production^8^ or through the stimulation of food‐intake.^3^ Consistent with an orexigenic function, Grosse et al^3^ reported a fall in INSL5 plasma levels after refeeding of starved mice; however, they observed poor reproducibility and batch dependence of several commercially available immunoassays. The detected plasma concentrations varied widely,^3^ but generally were in the range of 1–100 pg/mL, whereas a recent report detected ~100‐fold higher INSL5 levels in human serum.^9^ Importantly, however, with commercial immunoassays we were unable to detect INSL5 either in supernatants or lysates of primary colonic epithelial cultures that we routinely use to investigate secretion of GLP‐1 and PYY from L‐cells. As we were able to detect INSL5 in these cultures by immunohistochemistry,^3^ we aimed to develop an independent assay suitable for the investigation of stimulus secretion coupling in these *in vitro* systems.

Over the last two decades, mass spectrometry (in combination with electrospray ionization) has become a powerful tool in the analysis of peptides, both for quantitative analysis and for identification of potential new functional peptides. Studies have included the analysis of primary islet cell cultures where significant numbers of glucagon gene products were identified.^10^ More targeted mass spectrometry techniques using triple quadrupole systems have been employed to monitor peptides such as GLP‐1 7‐36 and 9‐36 in established colorectal cell lines.^11^ Furthermore, mass spectrometric approaches have proven to be sensitive enough to detect some gut hormone peptides at physiological (usually low pM or pg/mL) levels in circulating plasma.^12^, ^13^ Mass spectrometry has also been successfully applied to the detection and quantitation of peptides from the insulin/relaxin family. In contrast to most immunoassays, mass spectrometry can readily distinguish between peptides with only subtle differences as for example endogenous human insulin and the closely related Humalog, which is isobaric and differs only by a transposition of the proline and lysine residues at the C‐terminal end of the A‐chain.^14^ This study also demonstrated that mass spectrometric approaches can reach lower limits of quantitation in the region of 50 pg/mL in plasma for most of the insulin analogues. We thus investigated the possibility of using a MS‐based assay for INSL5 that would also allow the characterisation of the exact peptide sequence of stored and secreted products derived from the prepropeptide.

## 2 EXPERIMENTAL

### 2.1 Materials

All reagents were purchased from Sigma Aldrich (Poole, UK), except acetonitrile, methanol and trypsin that were purchased from Fisher Scientific (Loughborough, UK), Matrigel and Draq5 that were purchased from BD Bioscience (Oxford, UK) and Y27632 from Tocris Bioscience (Bristol, UK). Murine INSL5 peptide was purchased from Phoenix Pharmaceuticals (Burlingame, CA, USA).

### 2.2 Peptide extraction from FACS‐sorted murine L‐cells

Single cell suspensions of colonic epithelial cells were prepared with slight modifications to a previously described procedure.^15^ All animal procedures were approved by the University of Cambridge Animal Welfare and Ethical Review Body and conformed to the Animals (Scientific Procedures) Act 1986 Amendment Regulations (SI 2012/3039). The work was performed under the UK Home Office Project License 70/7824. Briefly, colons from 3–6‐month old mice expressing the reporter fluorophore Venus under the control of the glucagon promoter^16^ were harvested and washed with phosphate‐buffered saline (PBS). The external muscle layer was removed and tissue was incubated in PBS with 15 mM EDTA and 0.5mM dithiothreitol (DTT) for 10 min, transferred into cold PBS with Y27632 (10 μM) and shaken for 30 s to detach crypts. This was repeated five times and crypts and cell clumps detached from the tissue were centrifuged (300 *g*, 5min) after each step. Pooled sediments were digested into single cells in 0.25% trypsin‐EDTA for 5–10 min at 37°C. Single cells were washed and kept in cold HBSS with 0.1% BSA, Y27632 (10 μM), DAPI (1 μg/mL) and Draq5 (5 μM). Single live L‐cells were isolated based on forward and side scatter and Venus, DAPI and Draq5 fluorescence within 4 h using a FACSJazz (BD Biosciences, Singapore) at the Cambridge NIHR BRC Cell Phenotyping Hub. Peptides were extracted using a method adapted from a validated approach for enriching peptides from plasma,^17^, ^18^, ^19^ where a total of 6000–8000 positive cells were sorted and lysed directly into 800 μL of 80% ACN in water (v/v) in a Protein Lobind Eppendorf tube. Samples were centrifuged for 5 min at 10,000 *g* to pellet the proteins and the supernatant was transferred to a fresh tube and evaporated to dryness in an Eppendorf 5301 vacuum concentrator. Reduction and alkylation of disulphide bonds was performed (as described below) prior to LC/MS analysis, and 10 μL was injected onto the system.

### 2.3 Reduction and alkylation

The sorted cell peptide residue was reconstituted into 25 μL of 50 mM ammonium bicarbonate containing 10 mM DTT and heated at 60°C for 60 min. Then 5 μL of 100 mM iodoacetamide (IAA) in 50 mM ammonium bicarbonate were mixed in and the samples were stored in the dark for 30 min at room temperature and subsequently exposed to light for at least 30 min. 5 μL of 1% formic acid in water (v/v) was added prior to injection onto the LC/MS system.

### 2.4 Tissue homogenisation

Mice (3–6 months old) were sacrificed by cervical dislocation and the colon was excised. For human colon, fresh surgical specimens were obtained from the Tissue Bank at Addenbrooke's Hospital, Cambridge, UK, stored at 4°C and processed within a few hours of surgery. Work on human tissues was approved by the Research Ethics Committee under license number 09/H0308/24.

Tissue was washed with PBS and the outer muscle layer removed. Then 20–100 mg of tissue was minced and transferred into a fast prep lysing matrix D tube (MP Biologicals, Santa Ana, USA). Peptides were extracted from the tissue by adding 800 μL of 80% ACN in water (v/v) to the tissue pieces, and performing homogenisation using a Fast‐Prep 24 instrument (MP Biologicals) with four cycles of 40 s homogenisation (6m s^−1^). Precipitated proteins and tissue debris were pelleted by centrifugation (5 min at 10,000 *g*) and the supernatant transferred to Lobind tubes for evaporation at room temperature. All extraction steps were performed on ice or at 4°C to minimize peptide loss through enzymatic degradation and Lobind tubes were used throughout to counter adsorptive losses to plastic consumables. Dried extracts were reconstituted into 200 μL of 0.1% formic acid in water (v/v) and loaded onto a Waters HLB μElution solid‐phase extraction (SPE) plate, primed with 200 μL of methanol and 200 μL of reagent‐grade water before loading of the sample. Samples were washed with 200 μL of 0.1% formic acid in water (v/v) and 200 μL of 5% methanol & 1% acetic acid in water (v/v) before elution with 2 × 25 μL of 60% methanol in water with 10% acetic acid (v/v). For intact peptide studies, the extract was immediately diluted with 50 μL of 0.1% formic acid in water (v/v) prior to injection onto the LC/MS system. To confirm the sequence of the A and B chains, the SPE eluent (50 μL) was dried under oxygen‐free nitrogen (heated to 40 °C) on a SPE Dry evaporator system (Biotage, Upsalla, Sweden) prior to reduction and alkylation.

### 2.5 Nano LC/MS analysis of sorted cells and tissue homogenates

Peptide extracts were analysed using a Thermo Fisher Ultimate 3000 nano‐LC system coupled to a Q Exactive Plus Orbitrap mass spectrometer (ThermoScientific, San Jose, USA). The analysis was performed using nano‐flow‐based separation and electrospray approaches due to the low amount of material present in the samples. The extracts were injected onto a peptide trap column (0.3 × 5 mm; ThermoFisher Scientific) at a flow rate of 20 μL/min and washed for 10 min before switching in line with a nano easy column (0.075 × 250 mm; ThermoFisher Scientific) flowing at 300 nL/min. Both nano and trap column temperatures were set at 45°C during the analysis. The buffers used for nano‐LC separations were A: 0.1% formic acid in water (v/v) and B: 0.1% formic acid (v/v) in 80:20 ACN/water. Initial starting conditions were 2.5% B (equating to 2% ACN), and held for 5 min. A ramp to 50% B was performed over 145 min, and the column then washed with 90% B for 20 min before returning to starting conditions for 20 min, totalling an entire run time of 190 min. Electrospray analysis was performed using a spray voltage of 1.8 kV, the tune settings for the MS used an S‐lens setting of 70 v to target peptides of higher *m/z* values. A full scan range of 400–1600 *m/z* was used at a resolution of 75,000 before the top 10 ions of each spectrum were selected for MS/MS analysis. Existing ions selected for fragmentation were added to an exclusion list for 30 s.

### 2.6 Endogenous peptide identification

The acquired LC/MS files were searched using Peaks 8.0 software (Waterloo, ON, Canada) against the mouse and human Swissprot databases (Downloaded on 06‐May‐2016). A no‐digest setting was used, which enabled peptides of up to 65 amino acids in length to be matched, and precursor and product ion tolerances were set at 10 ppm and 0.05 Da, respectively. A fixed post‐translational modification of carbamidomethylation was applied to cysteine residues, whilst variable modifications included methionine oxidation, N‐terminal pyro‐glutamate, N‐terminal acetylation and C‐terminal amidation. A false discovery rate value of 1% was used to filter the results, with a minimum of 1 unique peptide also required.

### 2.7 Secretion of INSL5 from primary murine and human colon cultures

Following the identification of the reduced and alkylated A and B chains from INSL5 in the nano‐LC/MS/MS analysis, an assessment of the intact and secreted form of INSL5 was performed. Primary mixed epithelial cultures of human and mouse colon were generated using established methods.^4^, ^16^ Briefly, human and mouse tissues were washed and minced and digested with collagenase XI (respectively 0.5mg/mL and 0.4mg/mL) twice for 10 min and twice for 15 min. Crypts/cell clumps were collected and washed twice in culture medium (high‐glucose DMEM supplemented with 10% fetal bovine serum (FBS), 100 U/mL penicillin and 0.1 mg/mL streptomycin (P/S), and 2 mM L‐glutamine) before being plated on Matrigel‐coated plates. The culture medium was replaced 16–36 h after plating by washing with HEPES‐buffered saline ((mM): NaCl (138), KCl (4.5), HEPES (10), CaCl_2_ (2.6), MgCl_2_ (1.2), NaHCO_3_ (4.2), NaH_2_PO_4_ (1.2), pH adjusted to 7.4 with NaOH) with 0.001% BSA, diprotin A (30mM) and phosphoramidon (10μM). Subsequently, wells were incubated for 1 h at 37°C in this buffer (control) or in the additional presence of forskolin (10 μM), 3‐isobutyl‐1‐methylxanthine (IBMX, 10 μM) and glucose (10 mM). Supernatants were collected and cleared of cells and debris by centrifugation (5 min, 2000 *g*) and stored at −70°C until extraction. Samples were extracted using the SPE method described above, and were analysed without reduction and alkylation to assess the intact form of INSL5.

### 2.8 High flow rate analysis of primary colonic culture supernatants

A full scan analysis was performed on the extract using a high flow rate based LC/MS approach using a flow rate of 300 μL/min on the same instrumentation as described above. Solvents used for the separation were A: 0.1% formic acid in water (v/v) and B: 0.1% formic acid in ACN (v/v). A volume of 10 μL of extract was injected onto a HSS T3 UPLC™ column (2.1 × 50 mm; Waters, Elstree, UK) at a starting condition of 7.5% B, and eluted using a linear gradient up to 40% B over 16 min. The column was washed for 2 min at 90% B and returned to starting conditions for 2 min, totalling a run time of 20 min. A total of 40 μL of sample was injected onto the column for analysis. Mass spectrometry was performed using positive electrospray mode with a needle voltage of 3 kV, gas settings of 55 and 10 for sheath gas and aux gas flow rates. The temperature of the gas was set at 350°C and the transfer capillary at 350°C and a s‐lens value of 70 V. Full scan data was acquired over an *m/z* range of 500–1600, using a resolution of 70,000 and a maximum fill time of 200 ms. Tandem mass spectra were collected on the top 3 ions of each spectrum with a minimum *m/z* of 100, and a resolution of 17,500. Database searching was performed as mentioned earlier. The Phoenix Pharmaceutical INSL5 protein was characterised using this high flow rate analysis, where 10 μL of a 1 μg/mL standard was injected onto the LC/MS system.

### 2.9 Precision and accuracy of the SPE approach

An attempt was made to develop a quantitative methodology for the intact murine INSL5 peptide using a commercially sourced murine INSL5 peptide. However, the characterisation of the peptide demonstrated it was different from the endogenous peptide, and therefore could not be used to generate a fully quantitative methodology. The peptide was however used to assess the sensitivity, precision and accuracy of the SPE approach for a very closely related peptide to the endogenous protein. A calibration line of Phoenix INSL5 protein was generated in 0.001% BSA in 0.1% formic acid (v/v) over a range of 0.1–10 ng/mL. Seven calibration standards were generated (0.1, 0.25, 0.5, 1.0, 2.5, 5.0 and 10 ng/mL); 0.5 mL of each standard was extracted via the SPE method described above, and 40 μL of sample was injected onto the LC/MS system. Four levels of QC were generated (0.1, 0.3, 1 and 8 ng/mL) and extracted six times in order to assess the precision and accuracy of the analysis. The calibration line followed a quadratic fit, as no internal standard was available to be able to correct for ionisation or adsorptive losses during the extraction process. Recovery of the peptide from the BSA solution was assessed by analysing a spiked solution before and after extraction at a concentration of 8 ng/mL.

## 3 RESULTS AND DISCUSSION

### 3.1 Nanoflow‐LC/MS analysis of purified cells and homogenised tissue

**Figure 2:**
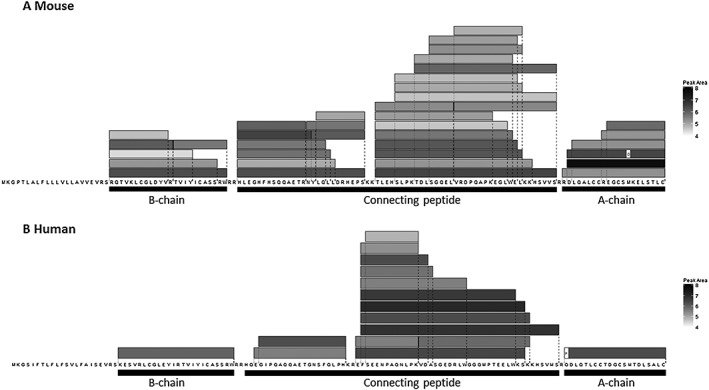
Peptides from the INSL5 prohormone detected in murine and human colonic samples

Initial analysis was performed on sorted colonic murine L‐cells as these are the main source of INSL5 in the body.^3^, ^20^ INSL5‐derived peptides were successfully detected after reduction/alkylation (Figure 2A). Interestingly, the extraction method was also sufficiently sensitive to detect INSL5‐derived peptides in crude tissue homogenates from both murine (not shown) and human colon (Figure 2B). The database search matched peptides throughout the entire INSL5 prohormone sequence except from the signal peptide (amino acid residues 1‐21), and three dibasic cleavage sites. These included the full‐length A and B chain peptides (and fragments of) and a significant number of peptides from the connecting‐peptide region. Interestingly, the Swissprot entry for the connecting peptide suggests that it is one single peptide (residues 49‐114 for both the mouse and human peptides); however, the data we have generated suggest that the connecting peptide is cleaved into two distinct peptides (and associated break down products) in both the murine and human tissues. We considered the true A and B chains to be the matched peptides that were present at the highest levels (based on peptide peak area), and shorter peptides were considered degradation products.

Each box represents a sequence detected and identified by LC/MS/MS that matches the mouse (top) or the human (bottom) INSL5 sequence and is represented above the corresponding sequence. Grey levels indicate the peptide peak area (log10 scale) from a representative experiment of mouse sorted colonic L‐cells and homogenates of human colonic tissue. Some post‐translational modifications to specific amino acids were detected and are indicated by a white box at the position of the modification: the methionine in the A chain was present in both a reduced and oxidised (O) form, and the N‐terminal of the human A chain only displayed the pyro‐glutamate form (P), as is well documented. The sequence specified in the Swissprot database is indicated by the black bars below the sequence.

The sequences of the A and B peptides with the highest peak area essentially matched the peptides specified at the Swissprot database. However, while the human A chain was confirmed to possess an N‐terminal pyroglutamate,^21^ we did not observe the N‐terminal arginine specified for the mouse A chain on Swissprot in whole tissue extracts. Instead the predominant A chain peptide started with the subsequent aspartate and only in extracts from FACS‐purified L‐cells could we detect an A chain with the N‐terminal arginine at an ~200‐fold reduced abundance compared to the predominant form. A likely explanation for these findings is that the N‐terminal arginine containing an A chain represents a processing intermediate and that this amino acid is removed in the mature murine INSL5. We also detected murine A chain peptides with an oxidised methionine in lysed FACS‐sorted cells (Figure 2A). The oxidised form of the A chain eluted well before the non‐oxidized form, therefore excluding an oxidation artefact during ionisation, but we cannot exclude the possibility that the oxidation occurs at other time points during sample processing rather than representing a true secreted hormone variant.

We also analysed a synthetic murine INSL5 purchased from Phoenix by MS/MS and confirmed the sequences given in the maunfacturer's data sheet. The A chain starts with the aspartate, similar to the predominant form we found endogenously produced in L‐cells. The B chain of the Phoenix peptide, however, incorporated an N‐terminal serine, which is thought to be part of the signal peptide as stated on Swissprot and accordingly appeared not to be present in the mature INSL5 peptide isolated or secreted from L‐cells.

**Figure 3:**
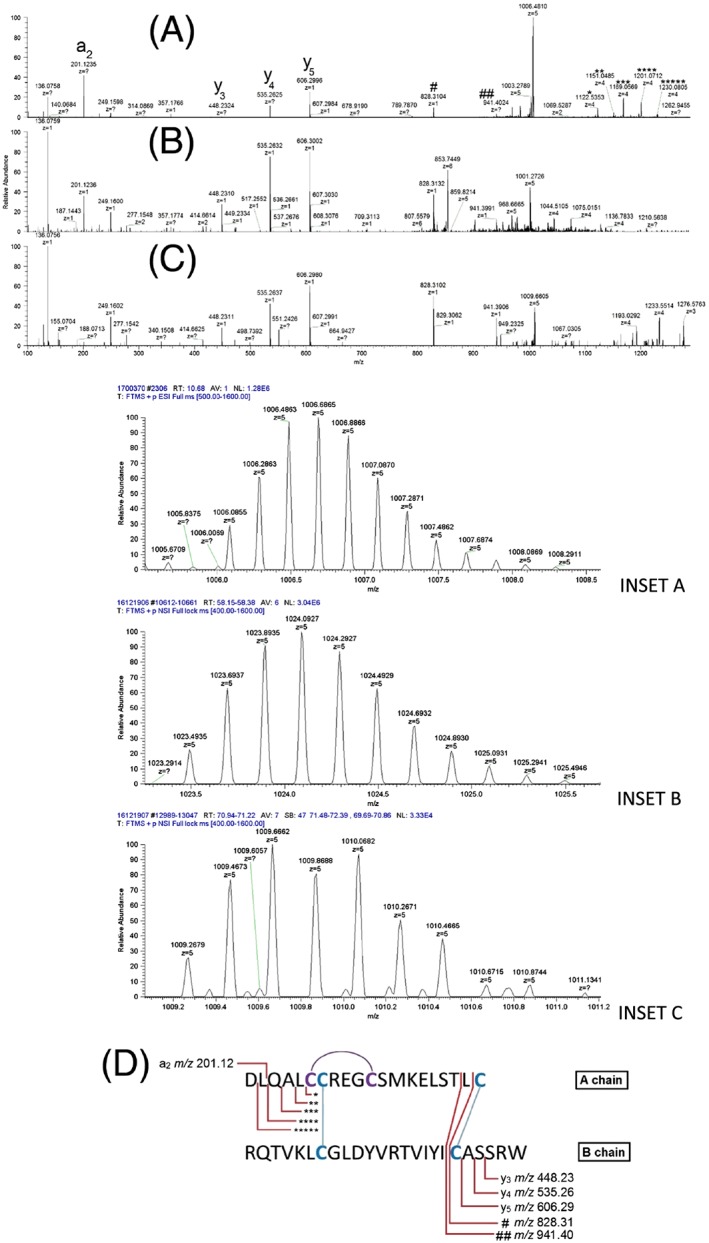
Product ion spectra of three INSL5 (A = mouse (from stimulated secretion sample), B = synthetic mouse (Phoenix), C = human (from stimulated secretion sample)), showing significant similarities in fragmentation patterns. The tandem mass spectrum of the Phoenix INSL5 [M+6H]^6+^ charge state was used as it generated a better fragmentation spectrum than the [M+5H]^5+^ charge state. Ions that are labelled in (A) indicate positive identifications of the fragmentation product represented in (D). The inset shows the [M+5H]^5+^ charge state for each peptide isoform, from which the intact peptides molecular weight was calculated. D, Proposed structure of mouse INSL5 and its fragmentation pattern [Color figure can be viewed at wileyonlinelibrary.com]

The combination of the multiple tissue sources in the LC/MS data and database searching results allowed us to propose a mature mouse INSL5 sequence (shown in Figure 3D) differing subtly from the predicted form indicated in the Swissprot database (Figure 1A) and the synthesised form commercially available from Phoenix (Figure 1B). Our data corroborates the sequence of endogenous human INSL5 that is present in the Swissprot database (Figure 1C). Mass spectrometric techniques have been applied to human INSL5 protein previously to characterise the prohormone cleavage sites after heterologous expression in a mammalian cell line (Cos7 co‐overexpressing furin), identifying an identical mature form to the one reported here.^5^ Whilst MS has been used to verify the sequence of recombinant and synthetically synthesised INSL5 variants,^22^ to our knowledge, no endogenously expressed INSL5 has previously been analysed using LC/MS.

### 3.2 LC/MS analysis of intact endogenous and synthetic INSL5 peptides

As INSL5 is supposed to be secreted from the colon, we assessed if we could detect the peptide in the supernatant of human and murine colonic primary cultures. Initially, we detected murine INSL5 in culture supernatants after reduction/alkylation, the A and B chain species resembling the ones observed in whole tissue lysates. Both human and mouse INSL5 could, however, also be detected intact, as can be seen in the insets of Figures 3A and 3C. The inset of Figure 3A shows the [M+5H]^5+^ charge state of the endogenous murine INSL5, which suggests that the peptide had a molecular weight that was calculated as 5025.412 Da, which is very close to the theoretical monoisotopic mass of the peptide with the sequence described in this study (5025.392, obtained from the chemical formula for murine INSL5 C_213_H_349_N_61_O_65_S_7_), and has an error of 3.97 ppm. The inset of Figure 3C shows the [M+5H]^5+^ charge state of the human INSL5, which suggests that the peptide had a molecular weight that was calculated as 5041.43 Da, which is very close to the theoretical monoisotopic mass of the peptide (5041.29, obtained from the chemical formula for human INSL5 C_213_H_341_N_57_O_70_S_7_), and has an error of 27 ppm. The fragmentation patterns of the [M+5H]^5+^ charge states of both analytes were investigated and both had several common fragmentation products, which were also seen with the synthetic Phoenix peptide standard (Figures 3A–3C). The synthetic murine INSL5 reference standard was obtained from Phoenix Pharmaceuticals to develop a quantitative LC/MS methodology, so that the levels of INSL5 could be measured in secretion samples. The initial LC/MS analysis of the intact Phoenix murine INSL5 peptide agreed with the certificate of analysis, and the data showed the standard contained a protein of 5112.47 Da, which, when compared to the theoretical molecular weight of this isoform of INSL5 of 5112.42, gave an accuracy of 9.7 ppm. The MS/MS fragmentation pattern of the peptide's [M+6H]^6+^ charge state was recorded and is displayed in Figure 3B. All fragmentation data was manually matched against theoretical fragments as the Peaks software was unable to match fragments of peptides that contain disulphide bonds.

Singly charged product ions were detected that corresponded to C‐terminal fragments of the B chain peptides up to the position of the disulphide‐bonded cysteines, whilst highly charged and large (y ion type) product ions were generated from fragmentation events at the N‐terminal region of the A chain. Fragments of the C‐terminal portion of the B chain were the most abundant, including the *m/z* 448.23, 535.26 and 606.29 ions which correspond to the y3, y4 and y5 fragments (Figure 3). The *m/z* 828.31 ion is believed to originate from a species that includes the C‐terminal cysteine residue of the A chain linked to the cysteine residue of the B chain and including the five other C‐terminal amino acids, therefore comprising a disulphide‐bonded fragment The expected *m/z* of this hypothetical A and B chain linked fragment is 828.31, which is identical to the experimentally acquired value (to 2 dp). An additional ion of m/z 941.40 was characterised as having this linked disulphide bond with either an additional leucine or isoleucine attached (Figure 3D). However, as these two amino acids are isobaric, the exact composition of the product ion cannot be confirmed. Other fragment ions that are present in the MS/MS spectrum appear to be derived from large and highly charged ([M+4H]^4+^ and [M+5H]^5+^ species) peptide fragments. These are likely fragmentation products of the peptide where N‐terminal residues are lost from the A chain peptide. Five specific *m/z* ions of interest are 1229.32, 1200.31, 1168.30, 1150.54 and 1122.28, which correspond to peptides which have masses of 4913.28, 4797.24, 4669.2, 4598.16 and 4485.16, respectively. Comparing these masses to the calculated intact molecular weight of the mouse INSL5 peptide (5025.44 Da) suggests that the peptide loses sequential masses of 112.13, 116.04, 128.04, 71.04 and 113.04 Da. The last three masses correspond well to the sequential loss of Q, A and L from the INSL5 A chain N‐terminus (DLQAL); however, the initial loss of the N‐terminal amino acids would be expected to give sequential mass differences of 115.09 Da (D) and 113.16 Da (L). This perhaps indicates that the N‐terminal of the A chain undergoes an alternative fragmentation procedure to the normal peptide backbone fragmentation pathway.

The mass spectrometric analysis of the Phoenix peptide suggests that the molecule was as sold; however, as mentioned previously, the peptide has an N‐terminal serine residue on the B chain, which differs from both the Swissprot specified sequence and the detected endogenous peptide. The Phoenix peptide standard did in fact contain a small amount of the endogenous peptide; however, this appeared to be present as an impurity, as its peak area was approximately 1.5% of that attributed to the Phoenix INSL5 with the additional N‐terminal serine residue. The difference in amino acid sequence between these two versions of murine INSL5 might not affect immunoassay‐based quantitation methods if the antibodies epitope is elsewhere in the INSL5 three‐dimensional structure. However, the addition of the serine residue means that mass spectrometric analysis approaches are compromised due to the significant change in the mass of the peptide. One potential approach to use the Phoenix peptide for quantitative studies is to reduce and alkylate the peptide, separating the two chains and analysing the A chain only, as this is identical to the endogenous version. However, using this approach would result in the loss of structural information, where any cleavages or modifications to the B chain would be lost if only the A chain is monitored.

### 3.3 Assessment of the precision and accuracy of the SPE method

**Figure 4:**
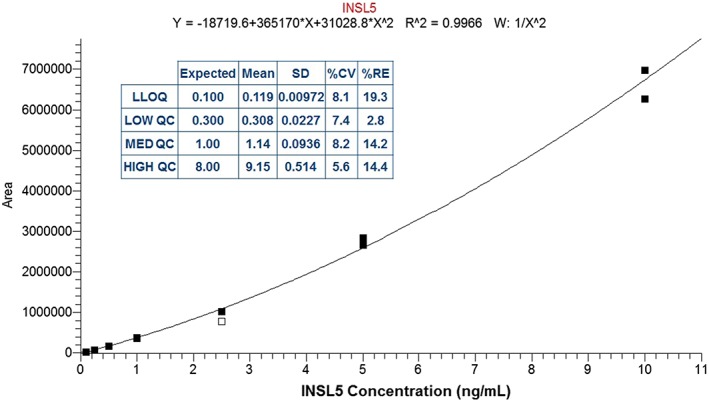
Calibration line of extracted Phoenix mouse INSL5 protein over the calibration range 0.1–10 ng/mL, demonstrating a quadratic fit. QC statistics of the precision and accuracy test are included as an inset (n = 6 at each level), showing good quantitative performance of the extraction method [Color figure can be viewed at wileyonlinelibrary.com]

The precision and accuracy of the SPE‐based LC/MS method was assessed over a concentration range of 0.1–10 ng/mL using the Phoenix peptide. Acceptance criteria for the analysis were 20% coefficient of variance (CV) and relative error (RE) for non‐LLOQ concentrations and 25% CV and RE at the LLOQ. These acceptance criteria are used for biological method validation, which we believe were appropriate for this method. The seven‐point calibration line demonstrated a quadratic fit, and resulted in a R^2^ value of 0.9966, where one of the 14 standards needed to be deactivated as it was outside the 20% acceptance criteria (Figure 4). The overall precision and accuracy values of the QCs can be seen in Figure 4, therefore demonstrating the method can generate good results across the selected calibration range.

The recovery of the extraction method was calculated as 59%; however, this demonstrated good reproducibility as the %CV of the five recovery assessments was less than 5.3%. This indicated that although the recovery was low, the reproducibility of the recovery was high, and suggested that further optimisation could potentially increase the sensitivity of the method through increasing the elution efficiency of the peptide from the SPE cartridges.

### 3.4 Quantification of INSL5 secretion in human and mouse colonic primary cultures

The primary colonic tissue cultures were incubated for 1 h with or without forskolin (10 μM), IBMX (10 μM) and glucose (10 mM), a cocktail well known to dramatically stimulate secretion of glucagon‐like peptide‐1 and peptideYY from L‐cells,^4^, ^16^ and the supernatant extracted using the SPE methodology. The murine and human incubations were pooled and then extracted in triplicate to assess the effect of these stimuli on the release of INSL5 from the tissues.

**Figure 5:**
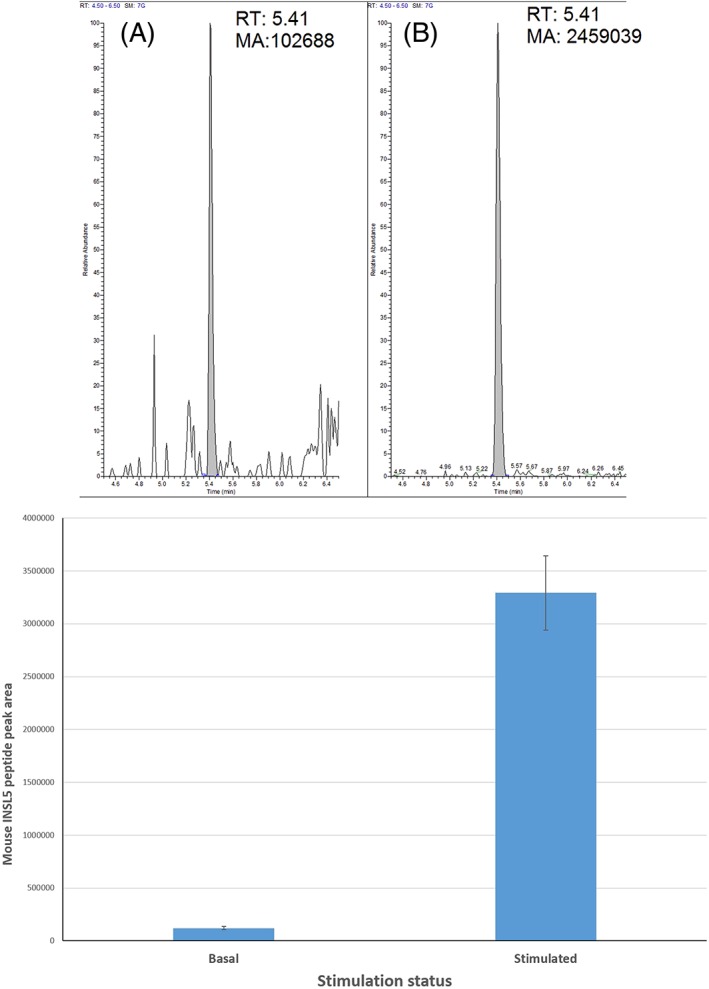
Example chromatograms of intact mouse INSL5 under basal conditions (A) and stimulated conditions (B) showing peak areas and relative noise levels. RT: Retention time; MA: measured area. The peptide peak areas (from four extractions of a pooled sample) demonstrate good reproducibility, and clear difference between the basal and stimulated states (C). Error bars indicate one standard deviation. The %CV of the four measurements was 13% and 11% for the basal and stimulated samples, respectively [Color figure can be viewed at wileyonlinelibrary.com]

The LC/MS analysis of the murine samples showed that the level of basal secretion was very close to the detection limit of the instrument; however, the peptide generated a peak that gave a signal‐to‐noise ratio of between 4 and 5. The stimulatory condition (forskolin/IBMX/glucose) increased the amount of INSL5 detected in the supernatant approximately 25‐fold in 1‐h incubations (Figure 5). The reproducibility of the SPE process on the culture supernatants was assessed by calculating the %CV of the endogenous INSL5 peptide peak area in the four measurements. These returned %CV values of 13% and 11% for the basal and stimulated samples, respectively, which are acceptable but could be improved further with internal standardisation approaches.

The analysis of the human primary culture supernatant failed to detect the peptide in any of the samples analysed using a high flow analysis. Therefore, the extract was re‐analysed using the described nano‐LC/MS approach, enabling the identification of INSL5 in the stimulated sample with a S/N ratio of ~20, generating a suitable MS/MS spectrum (Figure 3C). However, the signal from the basal sample was still too low to detect the peptide in its intact (disulphide‐bonded) form. Further experiments using tissue from a more distal region of the colon combined with reduction/alkylation of the extracts allowed us to detect INSL5 both in basal and stimulated conditions (data not shown). This suggests that further work is needed to improve the sensitivity of the methodology before it can be reliably applied to study INSL5 secretion from human‐derived cultures, but might also reflect that INSL5 content increases towards the more distal colon, whereas the exact source of human colonic tissue from surgery is somewhat varied. The possibility of protease‐derived losses of INSL5 from the human and murine secretion experiment is also a potential cause of sensitivity issues. Future method development will investigate the addition of protease inhibitors to the tissue culture supernatants (as long as they are compatible with cell function). However, we believe protease inhibitors were not needed in the ACN solvent in the sorted cell and homogenisation analysis, as proteases would be denatured in the 80% ACN solution, and not able to cleave peptides after extraction.

Further improvements to the human methodology might also involve using the Phoenix murine peptide as a surrogate internal standard for the human peptide, to account for potential losses during extraction. However, the Phoenix mouse INSL5 peptide is not an ideal surrogate internal standard for the endogenous murine peptide because of the low‐level impurity of the endogenous murine INSL5. Nevertheless, the data has shown that an LC/MS‐based methodology can be used to detect and measure endogenously produced murine INSL5 under different culture conditions, and allows comparative studies of the amount of INSL5 secreted. Due to the differences between the Phoenix INSL5 and the endogenous mouse peptide, absolute quantitation of the peptide was not possible. However, having in mind an unknown degree of error due to the mismatch between the Phoenix and the endogenous peptide sequence, quantification of the endogenous peptide peak relative to the Phoenix peak gave a rough estimate of 0.5–6.3 ng/mL for un‐ and stimulated conditions, respectively.

## 4 CONCLUSIONS

This study describes the adaptation of a well‐characterised extraction method for enriching peptides from complex matrices to the lysis of sorted enteroendocrine cells and whole tissue extracts to analyse INSL5 content. The initial sorted cell analysis enabled the detection and identification of the reduced and alkylated peptides from the gut hormone peptide INSL5. The analysis demonstrated good sensitivity, as fewer than 8000 cells were lysed in the analyses. The crude homogenisation approach also resulted in the detection of INSL5, albeit in the context of an increased background of other peptide fragments, derived from housekeeping proteins, such as histones and actin (data not shown), as might be expected from a tissue lysate in which the peptide of interest is only expressed in 1/1000 to 1/100 of the cells. The endogenous mouse peptide differed slightly from the Swissprot database reference sequence and we suggest this to be changed, as to the best of our knowledge this is the first report of a detailed sequence analysis of mature INSL5 from an endogenously expressing and processing source. The endogenously expressed and processed human peptide sequence by contrast is identical to the previous prediction from heterologous expression experiments.^21^ Furthermore, peptidomics analysis of the sorted cell extract and tissue homogenates showed that the connecting peptide was actually present as two distinct peptides due to the presence of a dibasic cleavage site within the amino acid sequence. This was apparent for both the murine and human connecting sequence, and differs from what is stated in the Swissprot database.

The focus of the method development then shifted from identification to quantitation on secreted intact INSL5. An SPE‐based methodology was developed that was capable of extracting INSL5 from the supernatant and concentrating it significantly, such that the intact peptide could be detected and quantified using a high flow rate analysis approach. Whilst a high flow rate analysis is less sensitive than nano‐LC/MS analyses, it is more robust and has significantly higher throughput. High flow analysis has been used previously to show how effective LC/MS/MS is as a quantitative tool for peptides and proteins, even generating comparable data against clinical analyser systems.^23^

Unfortunately, the Phoenix murine INSL5 that was purchased for generating a quantitative assay also demonstrated a different sequence from the endogenous peptide; therefore, future LC/MS quantitative strategies need to be focused first on obtaining a modified reference standard. Further, and significant, methodology developments will be required for detecting and quantifying INSL5 in plasma, as we expect these to be in the pM (pg/mL) range, similar the intestinal hormones GLP‐1 and PYY, which are co‐secreted from L‐cells. It is likely that in order to detect endogenous INSL5 in plasma, large plasma sample volumes could be required (not feasible in rodent models); however, nano‐LC/MS/MS and targeted SRM detection could achieve the required sensitivity with limited volume samples. Furthermore, the investigation of a hybrid immuno‐affinity LC/MS/MS analysis approach might be required, which was successfully employed to quantify circulating proglucagon products in human plasma.^13^

However, here we demonstrated an approach to measure secreted and cellular INSL5 levels in mouse and human samples that should enable further research investigating the production and secretion of this peptide recently implicated to play a role in metabolic homeostasis.
